# Effects of gastrokine-2 expression on gastric cancer cell apoptosis by activation of extrinsic apoptotic pathways

**DOI:** 10.3892/mmr.2014.2603

**Published:** 2014-09-30

**Authors:** LIN-SEN SHI, HAO WANG, FENG WANG, MIN FENG, MENG WANG, WEN-XIAN GUAN

**Affiliations:** Department of Gastrointestinal Surgery, The Drum Tower Clinical College of Nanjing Medical University, Nanjing, Jiangsu 210008, P.R. China

**Keywords:** gastric cancer, gastrokine-2, fas, apoptosis

## Abstract

Gastrokine-2 is a putative gastric cancer-specific tumor suppressor gene, the loss of which is known to be involved in the development and progression of gastric cancer, and restoration of gastrokine-2 expression inhibits growth of gastric cancer cells *in vitro*. However, the underlying mechanism of these effects requires elucidation. In the present study, expression patterns of gastrokine-2 protein were examined in gastric cancer tissues and cell lines. Expression of gastrokine-2 was restored in gastric cancer cells in order to assess its effect on cell viability, apoptosis and gene expression. A total of 76 gastric cancer tissues with corresponding normal mucosae samples, and two gastric cancer cell lines (SGC-7901 and AGS) were subjected to western blot analysis of gastrokine-2 expression. SGC-7901 cells were transiently transfected with gastrokine-2 cDNA and then treated with anti-CD95 and/or anti-Fas antibodies prior to analysis of cell viability, apoptosis and gene expression levels. Expression of gastrokine-2 protein was reduced or absent in gastric cancer tissues and gastric cancer cell lines. Following restoration of gastrokine-2 expression, the protein expression level of Fas was significantly increased, but no marked change was observed in the levels of bcl-2 and Bax proteins. Expression of gastrokine-2 protein reduced gastric cancer cell viability and induced apoptosis. Activity of caspase-3 and caspase-8 was increased, but caspase-9 activity remained unchanged in the SGC-7901 cells. Reduction or knockout of gastrokine-2 protein expression may contribute to gastric cancer development or progression, as the current study demonstrated that restoration of gastrokine-2 expression induces apoptosis of gastric cancer cells through the extrinsic apoptosis pathway.

## Introduction

Gastric cancer remains a significant worldwide health problem, as the fourth most common type of cancer and the second leading cause of cancer-related mortality worldwide (one million diagnoses of stomach cancer were made in 2008, with 740,000 related fatalities) (1–4). Although there has been a reduction in stomach cancer incidence in multiple countries, early detection remains the key to a better prognoses. However, in the early stages of gastric cancer, the majority of patients are asymptomatic and thus patients are commonly diagnosed at an advanced stage, leading to a low five-year survival rate (<10%) (4). The etiology of gastric cancer, similar to the majority of other types of cancer, remains to be defined, and the susceptibility of the individual to cancer may be altered by a combination of factors, including lifestyle and age, in addition to environmental and genetic aspects (5). For example, consumption of nitrate- or nitrite-rich food (grilled, salted or pickled foods) (6), presence of *Helicobacter pylori* infection (7), an age of >60 years and a history of stomach disorders or gastric cancer, have been reported to be possible variables that can lead to gastric cancer (8). By contrast, vitamin C, carotenoids and green tea have been implied to have preventive effects in gastric cancer (9). Furthermore, genetic susceptibility has been extensively investigated as an important contributor to inter-individual variation of gastric cancer risk (10). Accumulation of genetic and epigenetic changes (such as mutation and hypermethylation of tumor suppressor genes) has been confirmed to be involved in the development and progression of gastric cancer. A number of these genes, including p53, APC and c-erbB-2, are not gastric tissue-specific. The gastric tissue-specific genes may serve an essential role in the development and progression of gastric cancer. Thus, investigation of these genes may be useful to improve the understanding of the pathogenesis of gastric cancer, and to develop novel treatments.

The novel gastrointestinal tract-specific gene GDDR is abundantly expressed in normal gastric mucosae, but is downregulated or completely knocked out in gastric cancer (11). GDDR was originally cloned in our laboratory in 2002, by suppression-subtractive hybridization between the gastric carcinoma tissues and corresponding normal gastric mucosae and the ends-Marathon rapid amplification of cDNA ends (11). GDDR is a stomach-specific secreted protein and is a member of the gastrokine gene family. The GDDR protein is well-conserved and contains one BRICHOS domain with a pair of conserved cysteine residues, and is proposed to function in folding and intracellular transport or secretion (12). It possesses similarities to another gastric foveolar protein termed gastrokine-1 (13), thus GDDR has been renamed gastrokine-2 (14). Functionally, gastrokine-2 protein is involved in the replenishment of the surface lumen epithelial cell layer and maintenance of the mucosal integrity. Previous studies have demonstrated that expression of gastrokine-2 inhibits the proliferation of gastric cancer cells (15) and the progression of gastric cancer *in vivo*, in a trefoil factor 1-dependent manner (16,17). Thus, in the present study, the loss of expression of gastrokine-2 protein in human gastric cancer tissue samples was confirmed, and then a functional-grade purified anti-human CD95 (APO/Fas) antibody was used to activate, and an anti-Fas (human, neutralizing, clone ZB4) antibody was used to block the extrinsic pathway following transfection of gastrokine-2. The effects of gastrokine-2 protein on the regulation of gastric cancer cell viability and the underlying mechanism were investigated.

## Materials and methods

### Tissue samples

A total of 76 cancer and corresponding normal gastric tissues were collected from the Department of Gastrointestinal Cancer (The Drum Tower Clinical College of Nanjing Medical University, Nanjing, China) between November 2011 and June 2012. The clinicopathological characteristics of the patients with gastric carcinoma are outlined in Table I. All patients were pathologically confirmed to have gastric adenocarcinoma. The current study was approved by The Ethics Committee of The Drum Tower Clinical College of Nanjing Medical University. All patients or their legal guardians signed an inform consent form prior to participation in the study. Fresh tissue samples were obtained, snap-frozen using liquid nitrogen and stored at −80°C until use.

### Cell lines and culture

The SGC-7901 and AGS human gastric cancer cell lines were purchased from the Shanghai Institute of Cell Biology at the Chinese Academy of Sciences (Shanghai, China) and cultured in RPMI-1640 medium (Gibco, Carlsbad, CA, USA) supplemented with 10% fetal bovine serum (HyClone Laboratories, Logan, UT, USA), 1×10^5^ U/l penicillin and 100 mg/l streptomycin (CC033, Zhongke, Beijing, China)at 37°C in a humidified atmosphere with 5% CO_2_.

### Construction of expression vector and gene transfection

Human GDDR cDNA (Invitrogen, Carlsbad, CA, USA) was cloned into *Bam*HI/*Eco*RI restriction sites of the eukaryotic expression vector pcDNA3.1/Myc-His(+) (Invitrogen). Specifically, a primer (5′-GGAATTCTAATGAAAATACTTGTGGCAT-3′) containing a *Bam*HI linker in front of the initial GKN1 Met and 5′-CGGGATCCAACATGAATGTCTGCACAGA-3′ that abolished the GDDR stop codon for PCR amplification of the GDDR open reading frame were used. This PCR amplicon was then cloned into a pcDNA3.1/Myc-His(+) vector. Following sequence confirmation, the vector was termed pcDNA-GDDR. For gene transfection, the cells were subcultured and grown to the logarithmic growth phase then transiently transfected with pcDNA-GDDR or pcDNA3.1 (control) using Lipofectamine 2000 (Invitrogen), according to the manufacturer’s instructions. The transfection efficiency was evaluated by a parallel transfection using an EGFP vector (Invitrogen).

### Reverse transcription-polymerase chain reaction (RT-PCR)

SGC-7901 cells were divided into the control (Con), control vector-transfected (P) and GDDR cDNA-transfected (G) groups. At the end of experiments, total RNA (20–50 μg) was extracted from SGC-7901 human gastric cancer cells using TRIzol reagent (Invitrogen), and it was reverse transcribed into cDNA using an RNA PCR kit (DRR036A; Takara Bio, Inc., Otsu, Japan), according to the manufacturer’s instructions. These cDNA samples were amplified by PCR using a thermal cycler (Bio-Rad Laboratories, Hercules, CA, USA) with the following conditions: Initial denaturation at 94°C for 30 sec; followed by 40 cycles of 95°C for 5 sec, 65°C for 30 sec and 72°C for 30 sec; and a final extension at 72°C for 10 min. PCR fragments were separated by electrophoresis on a 1.5% agarose gel and visualized with ethidium bromide. Primer sequences (Invitrogen) were as follows: Forward: 5′-GACCCCTTCATTGACCTCAACTACA-3′ and reverse: 5′-GTCCACCACCCTGTTGCTGTAGCCA-3′ for GAPDH; forward: 5′-GTGGCATTTTGGTGGTG-3′ and reverse: 5′-CATTGTTGCTTGGGCTGA-3′ for GDDR; forward: 5′-AGACTGCGTGCCCTGCCAAGA-3′ and reverse: 5′-GGC CTGCCTGTTCAGTAACT-3′ for Fas; forward: 5′-GAGACA GCCAGGAGAAATCA-3′ and reverse: 5′-CCTGTGGAT GACTGAGTA-3′ for bcl-2; and forward: 5′-GACCCGGTG CCTCAGGATGC-3′ and reverse: 5′-GTCTGTGTCCAC GGCGGCAA-3′ for Bax.

### Protein extraction and western blot analysis

SGC-7901 cells were prepared as the Con, P and G groups. At the end of the experiments, total cellular protein was extracted from tissue specimens and gastric cancer cells using a lysis buffer containing 1X Protease Inhibitor Cocktail (Roche Diagnostics GmbH, Mannheim, Germany). Protein concentration was quantified using the Bicinchoninic Protein Assay kit (KeyGEN, China). Equal quantities of protein samples were resolved by 15% sodium dodecyl sulfate-polyacrylamide gel electrophoresis gels and electroblotted onto polyvinylidene fluoride membranes (Millipore, Billerica, MA, USA). The membranes were then blocked in 5% non-fat milk overnight, and the following day, membranes were incubated with a rabbit polyclonal anti-GDDR (ab70480, Abcam, Cambridge, UK), rabbit monoclonal anti-Fas (5709-1, Epitomics, Inc., CA, USA), anti-bcl-2 (BS1511, Bioworld, St. Louis, MN, USA), anti-Bax (BS2538 Bioworld) or anti-GAPDH (BSAP0063 Bioworld) for 4 h. Following washing with phosphate-buffered saline (PBS) with Tween-20 four times, and incubation with goat anti-rabbit secondary antibody (GAR0072, LiankeBio, Hangzhou, China) for 2 h at room temperature, the protein bands were visualized using an enhanced chemiluminescence kit (EMD Millipore, Billerica, MA, USA).

### Flow cytometry

SGC-7901 cells were prepared as Con, P and G groups, and then subjected to evaluation of Fas (also known as CD95) receptor expression. Briefly, cells (1–5×10^5^/100 μl) were scraped, subsequent to trypsin digestion without EDTA addition, washed twice with ice-cold PBS and the binding buffer, resuspended in the presence of an anti-human CD95 (APO-1/Fas) phycoerythrin (PE) antibody (12-0959, eBioscience, Inc., San Diego, CA, USA) and incubated in the dark for 30 min. The cell suspension was then washed with the binding buffer and resuspended in 200 μl binding buffer. For each sample, 2×10^4^ events were acquired by a CantoTM Flow Cytometer (BD Biosciences, Franklin Lakes, NJ, USA). The experiments were conducted in triplicate and repeated three times.

### Tumor cell viability assay

SGC-7901 cells were prepared as Con, P and G groups. A G+F group was created by coincubation of G group cells with a functional grade purified anti-human CD95 (APO/Fas; Epitomics, Burlingame, CA, USA) antibody at 5 mg/ml for 24 h. In the G+F+Z group, cells underwent the 48-h GDDR vector transfection plus coincubation with the CD95 (APO/Fas) antibody and an anti-Fas (human, neutralizing, clone ZB4 at 1 mg/ml; Merck Millipore, Darmstadt, Germany)] antibody. These cells were seeded into 96-well plates at 5×10^3^ cells/well and grown for up to 72 h. At the end of the experiments, 3-(4,5-dimethylthiazol-2-yl)-2,5-diphenyltetrazolium bromide (MTT; KeyGEN, Nanjing, China) at 100 μg/well was added to the cell culture, and the cells were incubated for another 4 h. A volume of 150 μl dimethyl sulfoxide (Sigma-Aldrich, St. Louis, MO, USA) was added to each well subsequent to removal of the supernatant. After shaking the plate for 20 min on a shaking board, cell viability was assessed by measuring the absorbance at 490 nm using an enzyme-labeling instrument (680 model; Bio-Rad Laboratories, Hercules, CA, USA). The experiments were conducted in quintuplicate and repeated three times. Growth inhibition (IR%) was calculated according to the following formula: IR% = [(the absorbance of blank control group-the absorbance of experimental group)/the absorbance of blank control group] × 100.

### Annexin V-fluorescein isothiocyanate (FITC) apoptosis assay

An Annexin V-FITC Apoptosis Detection kit with propidium iodide (eBioscience) was used to detect apoptosis. In brief, SGC-7901 cells were prepared as the Con, P and G groups. At the end of experiments, cells were harvested by centrifuging at 2,400 × g for 5 min, washed once in PBS, then once in 1X binding buffer, pelleted and resuspended at a concentration of 1×10^6^ in 100 μl of 1X binding buffer. A volume of 5 μl Annexin V-FITC was added to the cell solution, followed by incubation for 15 min at room temperature. It was then pelleted, washed with 1X binding buffer, and resuspended in 200 μl of 1X binding buffer. Next, 5 μl propidium iodide solution was added to the cells for a 15-min incubation at room temperature in the dark followed by the addition of 300 μl of 1X binding buffer. A minimum of 10,000 cells were subjected to flow cytometric analysis of the viable, apoptotic and necrotic cell populations. The results were quantified using Cell Quest software with FCS 2.0 files (Flowjo 7.6.5.1, BD Biosciences), according to the manufacturer’s instructions.

### Quantitation of caspase-3, -8 and -9 activity

SGC-7901 cells were prepared as the Con, P and G groups. At the end of the experiments, caspase activity was then measured using CaspGLOW Fluorescein Active Caspase-3, CaspGLOW Red Active Caspase-9 and CaspGLOW Red Active Caspase-8 Staining kits (#K183, #K199 and #K198, respectively; BioVision, Inc., Milpitas, CA, USA), according to the manufacturer’s instructions. Briefly, cells were resuspended in 300 μl complete growth medium at a concentration of 1×10^6^/ml, and incubated in a 37°C incubator for 45 min with the anti-caspase-3, -8 and -9 antibodies. The lysate was centrifuged at 4,800 × g for 5 min at 4°C, washed twice with the ice-cold wash buffer and the activity of caspase-3, -8 and -9 measured using the substrate peptides from the staining kits (FITC-DEVD-FMK, Red-IETD-FMK and Red-LEHD-FMK). The caspase activity was quantified by determining absorbance with the Multiskan Spectrum spectrophotometer (Thermo Fisher Scientific, Waltham, MA, USA) at Ex/Em = 540/570 nm. Analyses were performed in triplicate with at least three separate experiments.

### Statistical analysis

All experimental data were obtained from at least three independent experiments. The results are expressed as the mean ± standard deviation and were evaluated using one-way analysis of variance followed by Student’s t-test. Statistical analysis was performed using the SPSS 13.0 (SPSS, Inc., Chicago, IL, USA) for Windows software. P<0.05 was considered to indicate a statistically significant difference.

## Results

### Expression of gastrokine-2 in human gastric tissues and gastric cancer cell lines

**Figure 1:**
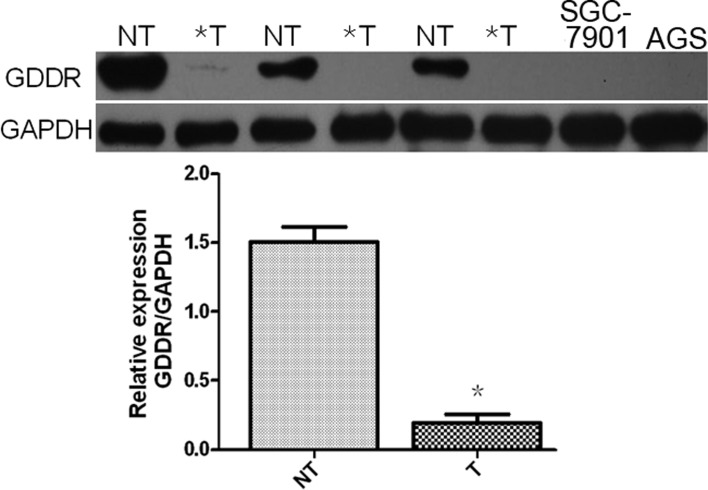
Expression of gastrokine-2 in gastric cancer and corresponding normal tissues and cell lines (SGC-7901 and AGS). Tissues specimens were collected and subjected to western blot analysis. ^*^P<0.01 vs. NT. NT, normal tissue; T, tumor tissue.

Gastrokine-2 expression was analyzed in 76 primary gastric cancer and corresponding normal tissues using western blot analysis. It was demonstrated that gastrokine-2 protein expression was reduced in 58 (84.0%) of the 76 cancer tissue samples compared with the corresponding gastric mucosal tissue samples (Fig. 1). Specifically, gastrokine-2 expression was reduced in 19 (73.07%), 32 (82.05%) and 7 (63.64%) of the 26 diffuse-, 39 intestinal- and 11 mixed-type gastric cancer samples, respectively. Expression of gastrokine-2 protein was indicated to be significantly lower in *H. pylori*-positive patients than the level in *H. pylori*-negative subjects (P<0.05; Table I), however, gastrokine-2 protein expression was not associated with tumor location, depth of invasion, lymph node metastasis, Lauren’s classification or tumor stage (P>0.05).

Gastrokine-2 expression was then analyzed in the two gastric cancer cell lines, and it was demonstrated its expression was absent in the SGC-7901 and AGS cells (Fig. 1).

### Restoration of gastrokine-2 expression in SGC-7901 gastric carcinoma cells

**Figure 2:**
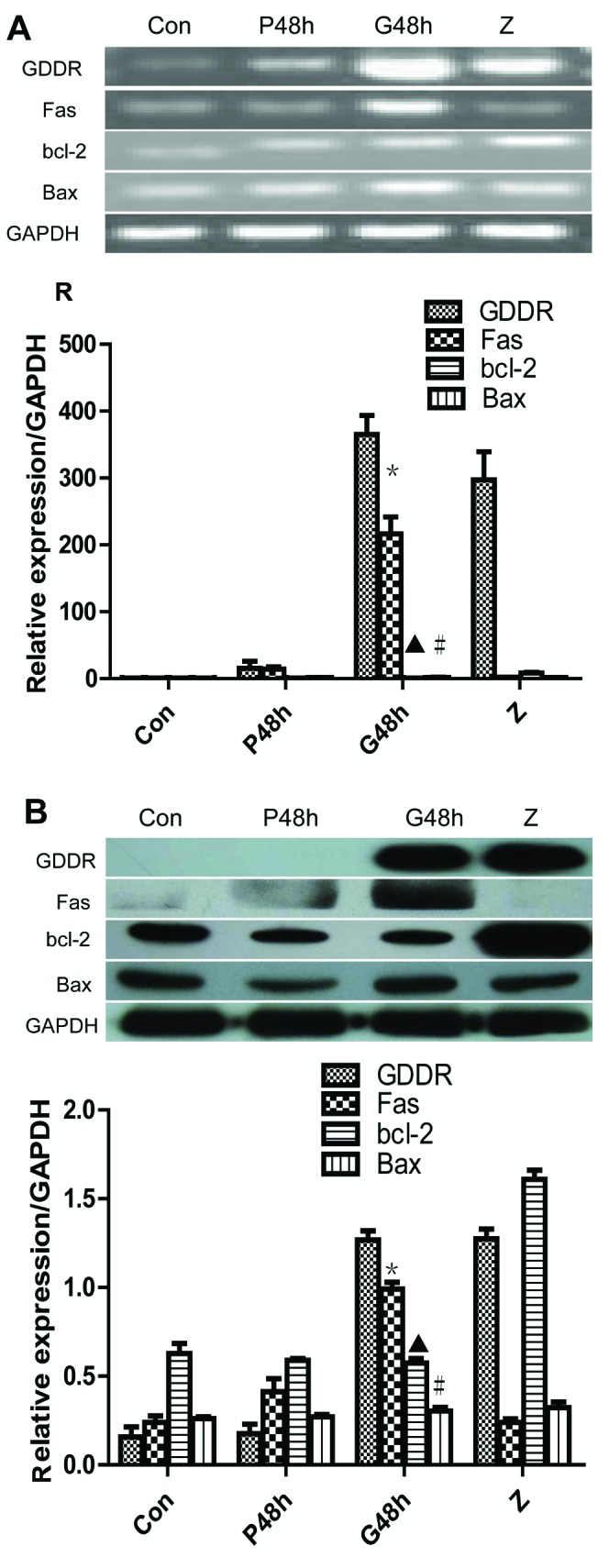
Effects of gastrokine-2 restoration on regulation of Fas, bcl-2 and Bax expression in gastric cancer SGC-7901 cells. Expression levels of Fas, bcl-2 and Bax (A) mRNA and (B) protein were evaluated following transfection of the cells with gastrokine-2 vector for 48 h (G48 h). ^*^P<0.05 vs. Con and P48 h; ^▲^P>0.05 vs. Con and P48 h; ^#^P>0.05 vs. Con and P48 h. Con, non-transfected SGC-7901 cells; P48 h, control vector; G48 h, gastrokine-2 vector; z, normal gastric tissue.

**Figure 3:**
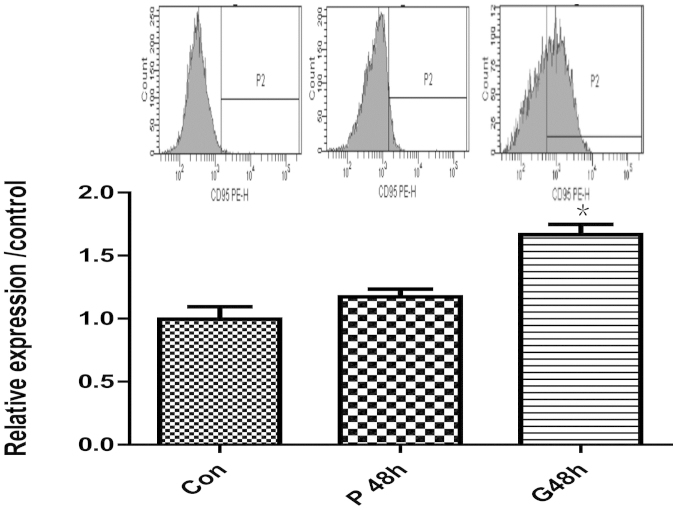
Flow cytometric analysis of Fas receptor expression in SGC-7901 cells. The cells were grown and transfected, with or without gastrokine-2 cDNA and then subjected to flow cytometric analysis of Fas expression using an anti-human CD95 antibody. ^*^P<0.05 vs. Con and P48 h. Con, non-transfected SGC-7901 cells; P48 h, control vector; G48 h, gastrokine-2 vector.

To determine the role of gastrokine-2 in gastric cancer cells, pcDNA3.1-GDDR or control pcDNA31 were transiently transfected into SGC-7901 cells. The results demonstrated that pcDNA3.1-GDDR restored gastrokine-2 expression levels in gastric cancer cells (Fig. 2A). The altered gene expression was then assessed, and it was indicated that the level of Fas mRNA was significantly upregulated 48 h after gene transfection (P<0.05 vs. non-transfected control and vector control; Fig. 2A). Fas protein level was also increased, as detected by western blot analysis (Fig. 2B) and flow cytometry (Fig. 3) with a rabbit monoclonal anti-Fas/CD95 and anti-human CD95 (APO-1/Fas) PE (Fig. 3). However, expression of bcl-2 and Bax mRNA and protein was not identified to significantly change from control levels.

### Restoration of gastrokine-2 expression reduces tumor cell viability in vitro

**Figure 4:**
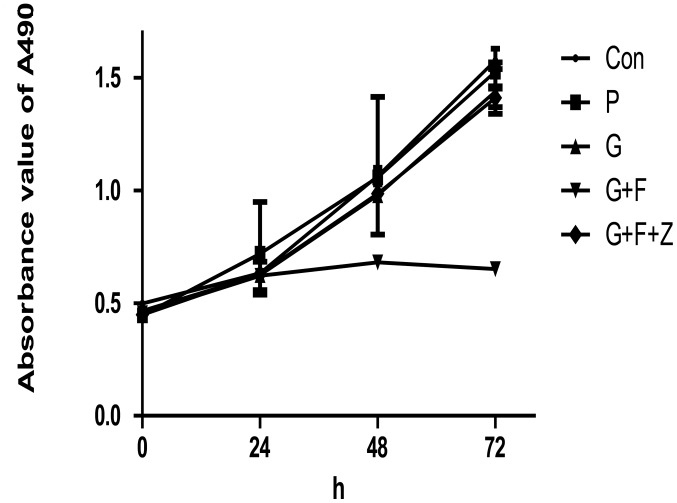
Effects of gastrokine-2 restoration on gastric cancer viability. SCG-7901 cells were grown and transfected with or without gastrokine-2 cDNA, and then subjected to an MTT assay. Cell viability was reduced in the G+F group (P<0.05 vs. Con, P, G and G+F+Z using analysis of variance). Con, non-transfected SGC-7901 cells; P, control vector; G, gastrokine-2 vector; G+F, gastrokine-2 vector + CD95 antibody; G+F+Z, gastrokine-2 vector + CD95 + Fas antibody.

Following transfection, the altered phenotypes of these gastric cancer cells was evaluated. A cell viability MTT assay was performed, and the results indicated that restoration of gastrokine-2 expression significantly reduced tumor cell viability in the monolayer culture. In brief, the inhibitory rate of G+F was 35.67±5.76 and 58.67±1.78% at 48 and 72 h, respectively. The P and G groups displayed reduced viability of 0.97±3.71 and 3±3.86%, and 13.69±2.29 and 7.72±5.28%, respectively (P<0.05). Additionally, the viability of G+F+Z cells was reduced compared with G+F cells (10.46±0.78 vs. 7.14±3.00% at 48 and 72 h, respectively; P<0.05; Fig. 4).

### Restoration of gastrokine-2 expression induces apoptosis in gastric cancer cells

**Figure 5:**
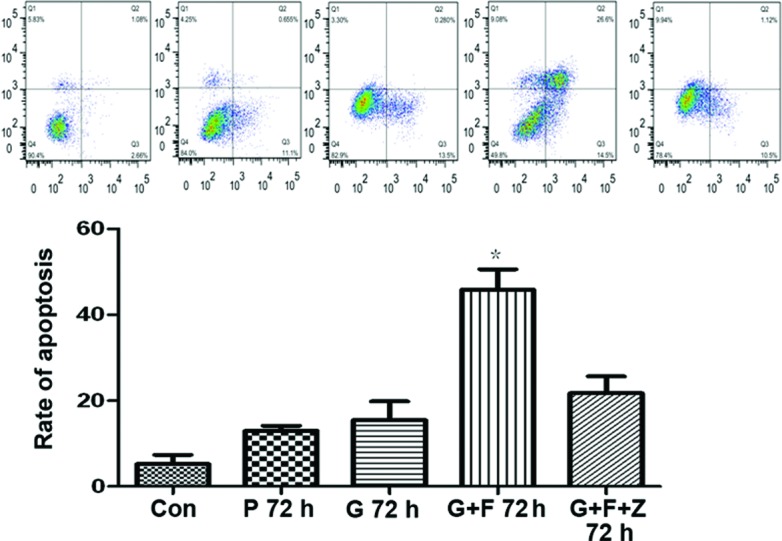
Effects of gastrokine-2 restoration on regulation of gastric cancer apoptosis. SCG-7901 cells were grown and transfected with or without gastrokine-2 cDNA and then subjected to flow cytometry assay. ^*^P<0.05 vs. Con, P72 h and G72 h. Con, non-transfected SGC-7901 cells; P72 h, control vector 72-h transfection; G72 h, gastrokine-2 vector 72-h transfection; G+F72 h, gastrokine-2 vector 48-h transfection + CD95 antibody 24-h incubation; G+F+Z, gastrokine-2 vector 48-h transfection + CD95 + Fas antibody 24-h incubation.

To assess the cause of the reduced cell viability, the rate of apoptosis was evaluated. Following 48-h gastrokine-2 transfection, SGC-7901 gastric cancer cells were incubated with functional grade purified anti-human CD95 (APO/Fas) for 24 h. The rate of apoptosis following antibody incubation was 45.89±8.20%, which was significantly higher than the level in cells transfected with gastrokine-2 vector (15.48±7.53%), control vector (12.97±1.99%), and non-transfected controls (5.24±3.71) (P<0.05). However, when the cells were coincubated with the two antibodies (CD95 and Fas; G+F+Z cells), the apoptosis rate was 21.71±6.90%, which was significantly reduced compared with the G+F cells (P<0.05; Fig. 5).

### Restoration of gastrokine-2 expression induces activation of caspase-3, -8, and -9

**Figure 6:**
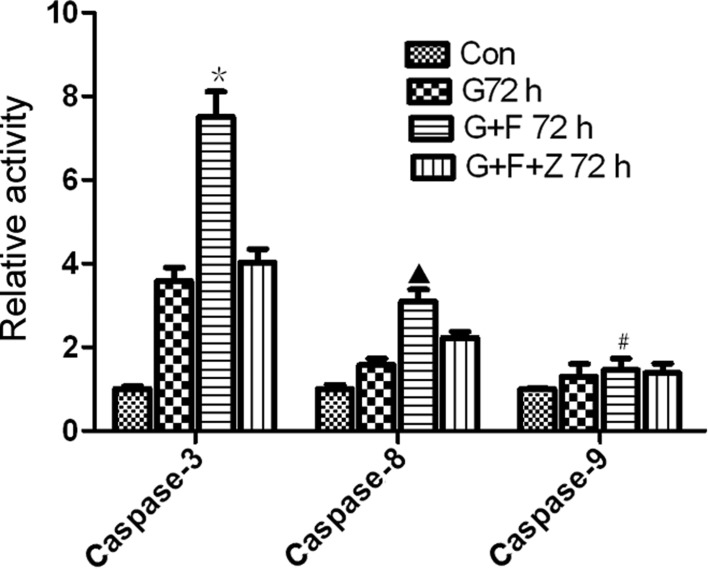
Effects of gastrokine-2 restoration on regulation of caspase activity. SCG-7901 cells were grown and transfected with or without gastrokine-2 cDNA and then subjected to caspase-3, -8 and -9 activity assays. ^*^P and ^▲^P<0.05 vs. Con, G 72 h and G+F+Z 72 h; ^#^P>0.05 vs. Con, G72 h and G+F+Z 72 h. Con, non-transfected SGC-7901 cells; G72 h, gastrokine-2 vector 72-h transfection; G+F72 h, gastrokine-2 vector 48-h transfection + CD95 antibody 24-h incubation; G+F+Z, gastrokine-2 vector 48-h transfection + CD95 + Fas antibody 24-h incubation.

To further assess the effect of gastrokine-2 restoration on the induction of apoptosis, the activity of caspase-3, -8 and -9 was determined. The data demonstrated that the relative activity of caspase-3 (7.5±1.04) and caspase-8 (3.09±0.49) was significantly higher in the G+F group compared with cells of the G group (3.58±0.57 and 1.58±0.26, caspase-3 and -8, respectively; P<0.05) and parental cell control (1.00±0.12 and 1.00±0.18 for caspase-3 and -8, respectively; P<0.01). The relative activity of caspase-3 (4.03±0.55) and caspase-8 (2.23±0.24) was lower in the G+F+Z group compared with the G+F group (Fig. 6). Furthermore, the activity levels of caspase-9 were 1.00±0.05, 1.03±0.11, 1.12±0.11, and 1.04±0.17 in the control, G, G+F and G+F+Z groups, respectively, indicating no significant differences (P>0.05; Fig. 6).

## Discussion

Apoptosis, also known as programmed cell death, is a basic biological process that functions to maintain homeostasis of the human body by removing undesirable cells (18). Apoptosis is controlled by a diverse range of cell signals, which can be classified into two major molecular signaling pathways; the extrinsic and intrinsic pathways (19–22). The extrinsic apoptotic pathway involves binding of the Fas ligand (FasL) to the Fas receptor (FasR; also termed CD95) (23,24), a transmembrane protein of the tumor necrosis factor family. This results in formation of the death-inducing signaling complex, which contains the Fas-associated death domain (FADD), caspase-8 and caspase-10. FADD is an adapter complex that recruits and activates caspase-8. Cleaved caspase-8 then induces cleavage and activation of executive caspase-3, and in turn, the activated capase-3 cleaves DNA molecules, leading to apoptosis (25–27). Alternatively, the intrinsic (or mitochondrial) pathway is largely dependent on the bcl-2 family of proteins (such as Bax) to induce cytochrome *c* release from the mitochondria. Cytochrome *c* binds to apoptotic protease activating factor-1, ATP and pro-caspase-9 to form a protein complex known as an apoptosome, in order to activate caspase-3 for induction of apoptosis. Different stimuli activate one of these apoptotic pathways, or both (23–27).

Previously, it has been demonstrated that the Fas/FasL pathway exerts a central role in induction of apoptosis, and alteration of this pathway has been observed in gastric adenocarcinoma cells (28). Gastric cancer tissues also indicated a downregulation of Fas, but increased FasL expression. Indeed, downregulation of Fas receptor expression in cancer cells can lead to apoptosis resistance and FasL stimulation (29,30). However, increased expression of FasL and reduced expression of caspase-3 in gastric cancer cells of the primary foci serve an important role in gastric carcinogenesis (27). FasL has also been implicated in de-differentiation, growth, invasion and metastasis of gastric cancer cells, through the induction of apoptosis in the infiltrating lymphocytes. By contrast, chemical substances derived from the primary foci of gastric cancer tissues and the metastatic microenvironment may inhibit the growth of metastatic cells by enhancing caspase-3 expression levels and decreasing those of FasL (27).

In the present study, the level of gastrokine-2 protein was reduced, or absent, in the majority of gastric cancer tissues and absent in two gastric cancer cell lines, which is consistent with the results reported by Du *et al* (11). Previous studies have not implicated gastrokine-2 as a putative gastric cancer-specific tumor suppressor gene (11). However, other studies have demonstrated that gastrokine-2 is a secretory peptide of human gastric surface mucous cells (31) and modulates gut epithelial cell proliferation (32). Gastrokine-2 expression has been reported to be attenuated in gastric adenocarcinomas (85% of diffuse and 54% intestinal type tumors) (33), whilst in gastric epithelial cells it has been indicated to be significantly upregulated following eradication of *Helicobacter pylori*, a risk factor for gastric cancer (34). These data indicated that gastrokine-2 may serve an important function in gastric epithelial cell homeostasis and that altered expression of gastrokine-2 protein may contribute to gastric carcinogenesis. Gastrokine-1, another member of the gastrokine family, has been demonstrated to introduce apoptosis in gastric cancer cells mainly through the Fas/FasL pathway (35).

The current study also demonstrated that restoration of gastrokine-2 protein expression upregulated Fas expression, but there was no significant difference in the expression level of bcl-2 and Bax, indicating that the extrinsic apoptosis pathway serves a role in gastrokine-2-induced gastric cancer cell apoptosis. To verify this, a functional grade purified anti-human CD95 (APO/Fas) antibody was used to promote apoptosis, and an anti-Fas (human, neutralizing, clone ZB4) antibody was used to block this extrinsic pathway. The data indicated that apoptosis was markedly increased in gastric cancer cells transfected with gastrokine-2 and incubated with functional grade purified CD95 (APO/Fas) antibody (48 h, 72 h), but the increase can be reversed by treatment with anti-Fas (human, neutralizing, clone ZB4) antibody. In order to further confirm this hypothesis, the activity of caspase 3, 8, and 9 was analyzed in these groups of gastric cell lines, and it was identified that caspase 3 and 8 in extrinsic apoptosis was activated or inhibited by functional grade purified CD95 (APO/Fas) and anti-Fas (human, neutralizing, clone ZB4) antibodies, respectively. However, caspase 9-related intrinsic apoptotic gene expression was not significantly altered.

In conclusion, to the best of our knowledge, the data from the current study demonstrated for first time that restoration of gastrokine-2 expression in SGC-7901 gastric cancer cells inhibits cell viability and induces apoptosis. Furthermore, it was demonstrated that apoptosis was induced through activation of the extrinsic apoptosis pathway. Following further investigation, gastrokine-2 may prove to be a potential target for novel molecular therapies for gastric cancer.

## Figures and Tables

**Table I:** Gastrokine-2 protein expression in gastric carcinoma.

	Gastrokine-2 protein expression		
		
Characteristic	+	−	P-value
Tumor location			0.699
Total	1	4	
Upper	7	23	
Middle	4	10	
Lower	6	21	
Depth of invasion			0.689
T0 or T1	2	4	
T2	2	3	
T3	10	43	
T4	4	8	
TNM stage			0.691
N0 (0)	3	10	
N1 (1–6)	6	11	
N2 (7–15)	4	11	
N3 (>15)	5	26	
Lauren’s classification			0.187
Intestinal	7	32	
Diffuse	7	19	
Mixed-type	4	7	
Tumor stage			0.667
I	1	4	
II	5	15	
III	12	35	
IV	0	4	
Anti-*H. pylori* IgG			0.039
+	4	36	
−	14	22	

TNM, tumor-node-metastasis. Upper, upper one third of the stomach; middle, middle one third of the stomach; lower, lower one third of the stomach.
